# Letter from J. Lee, M. D., D. D. S.

**Published:** 1858-11

**Authors:** J. Lee


CAMDEN, S. C., Sept. 1st, 1858.

Mr. JOHN T. TOLAND,

SIR:—I have your August number of the Dental Reporter, and cheerfully comply with your very moderate terms of subscription. As I can not repay you by ordering goods, I send you Post office stamps.

I see that C. P. Baird has nearly arrived at perfection in making swages for artificial dentures. If you will turn to the article "Model" in Harris' Dental Dictionary, you will see Lee's method on page 509, which for perfection of fit and expedition of construction, requires only to be known to supercede all others. I use the same metal for male and female casts.

I see you are down on Blandy's Cheoplasty, (and justly so.) His admirers and certifiers were certainly ignorant of the ancient Family Pill, which his antimonial plate will fully supercede.

The Pill I allude to was celebrated by Dr. J. R. Coxe, of the University of Pennsylvania, it consists simply of a bullet of antimony—this was swallowed by the member of the family requiring an aperient. Its oxydation produced a strong cathartic effect, it was watched for, well washed and put away for future use. Among the many virtues certified to be possessed by Blandy's Plates, they have neglected to praise its aperient powers, which is sometimes required by their not masticating their food well, and this to the great saving of expense for a variety of Patent Pills. I suppose you have seen the array of certificates published by Blandy. Of the names appended, some I know, some I know not.

I asked one who had certified to the cleanliness of the Plate, what he meant by it; his reply was, that it was easily cleaned, it was readily covered with oxide, but it was easily brushed off.

I agree with you, that men patenting a process in a profession which aspires to be liberal, benefit themselves neither in money or reputation; their patents are mostly humbugs, and they get money generally only from upstarts in the profession.

Blandy's alloy is essentially tin and antimony, for without antimony you can not make a full cast; the fine face of printer's types depend on the property of expansion in antimony, as it passes from the fluid form on cooling; this it does by crystalization, the lead giving it solidity.

Blandy's array of alloys is like the boy's "every thing at you," an expression used in playing marbles, and is only claimed in hopes an alloy may be made to answer the purpose.

Yours,

J. LEE, M. D., D. D. S.

CAMDEN, Oct. 1st, 1858.

MR. JOHN T. TOLAND,

DEAR SIR:—From the little I know of Blandy's Cheoplastic metal, I have no doubt a perfect fit may be made with it; but how any man at all acquainted with metallurgy can certify to such oxidable compounds remaining pure in the mouth, where 18 carat gold readily tarnishes, I can not understand.

You ask to publish the letter written to you. I have no copy of it, still if you think any useful end can be accomplished by it in putting down quackery in Dentistry, with however high names it may be foisted on the community, I have no objection, as I certainly in it said nothing I did not know or believe.

The reference I made to my mode of making swages for stamping gold plate was right as to the paging of the 1st edition, I have no subsequent edition by me. I will copy it, its very simplicity may have caused it to be left out, but those who try it will not readily give it up.

